# The feasibility of using liver function indices and FibroScan in combination to predict the occurrence of anti-tuberculosis drug-induced liver injury in Patients with liver disease

**DOI:** 10.5937/jomb0-50878

**Published:** 2025-01-24

**Authors:** Wu Shaoqiang, Yang Qiaohua, Li Huai, Li Yongzhong

**Affiliations:** 1 Hunan University of Medicine General Hospital, Infectious Disease Center, Department of Hepatology, Huaihua, China

**Keywords:** liver function indices, FibroScan, anti-tuberculosis drug-induced liver injury, liver disease, nomogram, indeksi funkcije jetre, FibroScan, oštečenje jetre izazvano anti-tuberkuloznim lekovima, bolest jetre, nomogram

## Abstract

**Background:**

The study aims to assess the feasibility of using a combined approach of liver function indices and FibroScan measurements as a predictive tool for the early detection of anti-tuberculosis drug-induced liver injury (DILI) in patients with existing liver disease.

**Methods:**

A retrospective cohort study was conducted, including adult tuberculosis patients with documented liver disease. Liver function was assessed using standard biochemical parameters, and FibroScan examinations were performed to determine liver stiffness measurement (LSM). Patients were monitored for clinical and biochemical signs of DILI throughout treatment. Logistic regression models and Receiver Operating Characteristic (ROC) curves were used for data analysis. Statistical significance was set at P<0.05.

**Results:**

Patients who developed DILI showed significantly higher levels of ALT, AST, total bilirubin, GGT, and LSM, with strong positive correlations between these markers and DILI occurrence. Logistic regression analysis revealed elevated ALT, AST, TBIL, and GGT were strongly associated with an increased likelihood of DILI. The area under the ROC curves indicated excellent predictive accuracy of these parameters. A nomogram for predicting DILI based on the combined biomarkers was established.

**Conclusions:**

The study demonstrates the feasibility of combining liver function indices and FibroScan measurements to predict anti-tuberculosis DILI. The results highlight the importance of baseline liver health assessment and offer promising implications for clinical practice, aiding in individualized risk estimation and therapeutic decision-making for patients with liver disease initiating anti-tuberculosis therapy. Further validation in larger cohorts is warranted to strengthen the predictive model.

## Abstract (sr)

**Uvod:**

Studija ima za cilj da proceni izvodljivost korišćenja kombinovanog pristupa indeksa funkcije jetre i merenja FibroScan uređajem kao prediktivnog alata za rano otkrivanje oštećenja jetre izazvanog anti-tuberkuloznim lekovima (DILI) kod pacijenata sa postojećim bolestima jetre.

**Metode:**

Sprovedena je retrospektivna kohortna studija koja je obuhvatila odrasle pacijente sa tuberkulozom i dokumentovanom bolešću jetre. Funkcija jetre je procenjivana korišćenjem standardnih biohemijskih parametara, a FibroScan pregledi su izvršeni kako bi se odredila mera fibroze jetre (LSM). Pacijenti su praćeni zbog kliničkih i biohemijskih znakova DILI tokom celokupnog tretmana. Za analizu podataka su korišćeni logistički regresioni modeli i ROC krive (Receiver Operating Characteristic). Statistička značajnost je postavljena na P<0,05.

## Introduction

Tuberculosis (TB) remains one of the most significant infectious diseases worldwide, with considerable morbidity and mortality [1] [2]. The standard treatment regimen for tuberculosis involves using various antitubercular drugs, some of which possess hepatotoxic potential [3] [4]. Drug-induced liver injury (DILI) is a major adverse event affecting patient compliance and treatment success. Patients with preexisting liver disease are at an increased risk of developing hepatotoxicity due to the compromised metabolic capacity of the liver [5] [6].

The identification and prediction of anti-tuberculosis drug-induced liver injury in this vulnerable group is crucial for early intervention and the prevention of severe liver damage [3]. In this context, non-invasive liver function indices and elastography-based FibroScan measurements have emerged as promising tools for assessing hepatic health [7] [8] [9] [10]. Liver function tests (LFTs) are a standard set of blood tests used to provide information about the state of a patient’s liver [11]. These tests include measurements of enzymes and proteins such as alanine aminotransferase (ALT), aspartate aminotransferase (AST), alkaline phosphatase (ALP), and bilirubin levels. Fibro-Scan, on the other hand, applies transient elastography to measure liver stiffness, an indirect marker of fibrosis and cirrhosis [5] [3] [12].

The prospective use of a combination of these modalities to predict the risk of anti-tuberculosis DILI has the potential to tailor therapy approaches and improve patient outcomes [13] [14]. Yet, the heterogeneity in the progression of liver disease and differing mechanisms of drug toxicity pose challenges in the establishment of a comprehensive predictive model [8].

This study aims to explore the feasibility of using a combined approach of liver function indices and FibroScan measurements as a predictive tool for the early detection of anti-tuberculosis drug-induced liver injury in patients with existing liver disease. By analyzing the relationship between baseline liver health parameters and treatment outcomes, we propose developing a predictive framework that healthcare professionals can utilize to stratify risk and decide on the most appropriate therapeutic interventions.

## Materials and methods

### Study design and participants

This retrospective cohort study included tuberculosis patients who received standard antitubercular therapy at a tertiary care centre between October 2022 and October 2023. Inclusion criteria encompassed adult patients (18 years or older) with a confirmed diagnosis of TB who had a documented liver disease (as evidenced by medical history and liver function tests). Exclusion criteria included patients with acute hepatic failure, hepatocellular carcinoma, co-infection with HIV, or previous history of DILI due to drugs other than antitubercular agents.

### Liver function assessment

Liver function was assessed using standard biochemical parameters, including serum ALT, AST, ALP, and total bilirubin. Blood samples were collected within a week before initiating antitubercular therapy and subsequently at two weeks, one month, and two months after initiation of the therapy. Serum enzymes and bilirubin levels were measured using an automated clinical chemistry analyzer (Yste180c, Guangzhou Yueshen Medical Equipment Co., Ltd, Guangzhou, China).

### Transient elastography

FibroScan examination (Echosens, Paris, France) was performed to determine liver stiffness measurement (LSM) as an indirect marker of liver fibrosis. Certified hepatologists performed the examination without knowing the clinical data. Measurements were taken at baseline and at the end of the second month of antitubercular therapy. A 10-MHz transducer was used, and the median value of ten successful measurements was recorded for each patient.

### Monitoring and definition of DILI

Patients were monitored for clinical and biochemical signs of DILI throughout the course of treatment. DILI was defined according to the International DILI Expert Working Group, with an elevation in ALT or AST levels greater than three times the upper limit of normal (ULN) with symptoms or an increase greater than five times ULN without symptoms, in conjunction with increased total bilirubin levels more than twice the ULN.

### Data analysis

Descriptive statistics were used to summarize the baseline characteristics. For inferential analysis, logistic regression models were utilized to determine the predictive value of baseline liver function tests and FibroScan scores for developing DILI. Receiver Operating Characteristic (ROC) curves were constructed to assess the model’s predictive accuracy. All statistical analyses were conducted using SPSS 22.0 Statistics software (IBM Corp., Armonk, NY, USA). Normally distributed data were expressed as mean±standard deviation (SD) and analyzed using the t-test. All statistical tests were two-tailed, and *P*<0.05 was considered statistically significant.

## Results

### Baseline characteristics

The baseline characteristics of the study population are summarized in Table 1. The mean age of participants in the DILI group was 42.65±10.15 years, while in the non-DILI group, it was 41.21±8.25 years. No significant difference in age was observed between the groups (*P*>0.05). Gender distribution also showed no significant difference between the two groups, with 39 males and 26 females in the DILI group and 42 males and 26 females in the non-DILI group (*P*>0.05). Liver cirrhosis was comparable in both groups, as were the incidences of HBV and HCV (*P*>0.05). Additionally, the use of concomitant medications such as statins and NSAIDs (*P*>0.05) did not differ significantly between the DILI and non-DILI groups.

**Table 1:** Baseline Characteristics of the Study Population.

Variable	DILI (n=65)	Non-DILI (n=68)	t/χ^2^	*P*
Age (years)	42.65±10.15	41.21±8.25	0.898	0.371
Gender (Male/ Female)	39/26	42/26	0.001	0.975
Liver Cirrhosis	21	22	0	1
HBV	15	14	0.019	0.891
HCV	13	14		1
Concomitant Medications (Statins)	17	18	0	1
Concomitant Medications (NSAIDs)	7	7	0	1

### Liver function markers and FibroScan LSM

The comparison of liver function markers and FibroScan measurements between DILI and non-DILI patients is detailed in Table 2. Significantly higher levels of ALT (80.24±10.83 U/L), AST (70.08±8.38 U/L), total bilirubin (2.05±0.53 mg/dL), and GGT (40.37±5.35 U/L) were observed in DILI patients compared to non-DILI patients. Conversely, non-DILI patients exhibited significantly lower levels of these markers: ALT (30.37±5.38 U/L), AST (25.27±3.46 U/L), total bilirubin (1.05±0.21 mg/dL), and GGT (20.18±3.38 U/L). Albumin levels were also significantly lower in DILI patients (3.25±0.43 g/dL) than in non-DILI patients (4.09±0.31 g/dL). Furthermore, LSM via FibroScan was notably higher in DILI patients (8.55±1.25 kPa) compared to non-DILI patients (5.01±0.83 kPa). All differences were found to be statistically significant, with p-values less than 0.001 and large t-values, illustrating distinctive differences in these markers between the two patient groups.

**Table 2:** Comparison of Liver Function Markers and FibroScan LSM in DILI and Non-DILI Patients.

	ALT (U/L)	AST (U/L)	TBIL (mg/dL)	ALB (g/dL)	GGT (U/L)	LSM (kPa)
DILI	80.24±10.83	70.08±8.38	2.05±0.53	3.25±0.43	40.37±5.35	8.55±1.25
Non-DILI	30.37±5.38	25.27±3.46	1.05±0.21	4.09±0.31	20.18±3.38	5.01±0.83
t	33.39	39.974	14.144	12.934	25.894	19.1
*P*	<0.001	<0.001	<0.001	<0.001	<0.001	<0.001

### Correlation analysis

The results of the correlation analysis between liver function markers/FibroScan LSM and the occurrence of DILI are shown in Table 3. ALT levels demonstrated a strong positive correlation with DILI (r=0.947, *P*<0.001), explaining 89.7% of the variance (R2=0.897). Similarly, AST levels were strongly correlated with DILI (r=0.963, *P*<0.001), with 92.6% variance explained (R2=0.926). Total bilirubin levels were also positively correlated with DILI occurrence (r=0.782, *P*<0.001), accounting for 61.2% of the variation. Albumin levels exhibited a moderate negative correlation with DILI (r=-0.751, *P*<0.001), accounting for 56.4% of the variation. In addition, GGT levels showed a strong positive correlation with DILI (r=0.916, *P*<0.001), explaining 83.9% of the variation. All correlations were statistically significant (*P*<0.001), indicating that higher levels of ALT, AST, total bilirubin, and GGT were associated with increased likelihood of DILI, while higher albumin levels predicted lower risk.

**Table 3:** Correlation Analysis between Liver Function Markers or FibroScan LSM and DILI.

	ALT (U/L)	AST (U/L)	TBIL (mg/dL)	ALB (g/dL)	GGT (U/L)
r	0.947	0.963	0.782	-0.751	0.916
R^2^	0.897	0.926	0.612	0.564	0.839
*P*	<0.001	<0.001	<0.001	<0.001	<0.001

### Logistic regression analysis

The logistic regression analysis for Anti-TB DILI is presented in Table 4. The results reveal that ALT, AST, TBIL, and GGT coefficients have statistically significant positive associations with the occurrence of DILI. Specifically, for every unit increase in ALT, the odds of DILI increased by 52.655 times; for AST, this increase was by 7.977 times. Total bilirubin also showed a substantial association, with an odds ratio of 6584.543 for every unit increase. However, for every unit increase in albumin, the odds of DILI decreased by 0.002 times. GGT also exhibited a significant positive association with DILI, with an odds ratio of 17.7 for every unit increase. These results indicate that higher levels of ALT, AST, TBIL, and GGT are associated with an increased likelihood of DILI, while higher levels of albumin predict a lower risk of DILI.

**Table 4:** Logistic Regression Analysis for Anti-TB DILI.

	ALT (U/L)	AST (U/L)	TBIL (mg/dL)	ALB (g/dL)	GGT (U/L)	ALT (U/L)
coef	3.964	2.077	8.792	6.034	2.874	4.517
odds ratio	52.655	7.977	6584.543	0.002	17.7	91.578
B	0.002	0.001	4.95	6.088	0.793	3.528
beta	3.964	2.077	8.792	-6.034	2.874	4.517
AIC	4	4	58.28	82.081	8.212	27.675
BIC	9.781	9.781	64.06	87.862	13.992	33.456
*P*	0.998	0.999	<0.001	<0.001	0.428	<0.001

### ROC analysis

The ROC analysis for DILI prediction is summarized in Table 5. The sensitivity values for ALT and AST were 1, indicating a strong ability to identify true positives correctly. Total bilirubin displayed a high sensitivity of 0.954, while albumin showed a sensitivity of 0.815, signifying a slightly lower but still substantial ability to detect true positives. GGT had a sensitivity of 1, demonstrating its robustness in correctly identifying positive cases. In terms of specificity, all the markers showed high values, with GGT and AST having the highest specificity of 0.985 and ALT showing a specificity of 1. The area under the curve (AUC) values indicated excellent discriminative ability for all markers, particularly GGT, which had an AUC of 1. The Youden Index, representing the overall effectiveness of each marker, underscored their robustness in predicting DILI, with GGT showing the highest Youden Index of 0.985. These results collectively highlight the strong predictive capability of these markers, particularly GGT, in identifying DILI cases.

**Table 5:** ROC Analysis for DILI Prediction.

	ALT (U/L)	AST (U/L)	TBIL (mg/dL)	ALB (g/dL)	GGT (U/L)	ALT (U/L)
sensitivities	1	1	0.954	0.815	1	0.969
specificities	1	1	0.926	0.956	0.985	0.985
auc	1	1	0.969	0.947	1	0.993
youden index	1	1	0.88	0.771	0.985	0.955

### Nomogram

**Figure 1:**
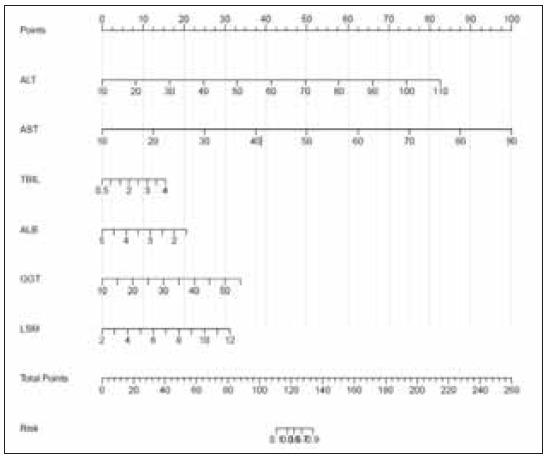
Nomogram for predicting DILI.

Nomograms can incorporate more predictors without sacrificing clarity, making them more comprehensive compared to the binary nature of ROC analysis. By allowing a more individualized and comprehensive risk prediction approach, nomograms are a valuable tool in clinical decision-making. The nomogram for predicting DILI using all involved biomarkers is shown in Figure 1.

## Discussion

This study aimed to explore the feasibility of using a combination of liver function indices and FibroScan measurements as a predictive tool for anti-tuberculosis drug-induced liver injury (DILI) inpatients with pre-existing liver disease. Our results demonstrate that baseline levels of liver enzymes, bilirubin, albumin and GGT, as well as FibroScan liver stiffness measurements, differ significantly between those who developed DILI and those who did not during anti-TB treatment. Correlation and logistic regression analyses revealed that elevated ALT, AST, TBIL, GGT, and LSM were strongly associated with increased risk of DILI. Furthermore, the area under the ROC curves indicated excellent predictive accuracy of these parameters. Finally, a nomogram for predicting DILI was established.

There are several possible explanations for these findings. Elevated baseline liver enzymes and bilirubin reflect compromised hepatic reserve in patients with underlying liver dysfunction [15] [16] [17]. This leaves little metabolic capacity to counteract the hepatotoxic effects of anti-TB drugs [18]. Higher FibroScan scores implicate advanced fibrosis or cirrhosis, impairing the normal detoxification pathways of the liver. GGT, a marker of cholestasis, may indicate disrupted biliary excretion of toxic metabolites [19] [20] [21]. Albumin, a negative acute phase reactant, inversely correlates with the degree of hepatic necroinflammation [22] [23]. Thus, patients with deranged baseline liver tests and elastography have diminished hepatic compensatory abilities, predisposing them to drug-induced toxicity.

The current study highlights the importance of assessing baseline liver health prior to initiating hepatotoxic drugs in vulnerable patients. A combined evaluation of biochemical and elastographic parameters can effectively discriminate high-risk from low-risk groups. This may aid therapeutic decision-making, such as individualizing drug regimens, instituting closer monitoring, or considering alternative treatment options. Furthermore, the nomogram developed based on our predictive model can offer clinicians an intuitive and personalized risk estimation tool at the bedside. Early identification of at-risk patients through such predictive strategies could help mitigate adverse outcomes and improve treatment success rates for tuberculosis.

Coupled with the logistic regression analysis, building a nomogram to comprehensively predict the likelihood of DILI based on the combined influence of these markers offers a personalized and intuitive tool for risk estimation in clinical settings [24] [25]. Nomograms excel in presenting complex predictive models in a user-friendly graphical format, allowing for a more individualized risk assessment that could aid in clinical decision-making [26]. Healthcare providers can visually interpret and compute the overall risk estimate through the nomogram by considering multiple liver function markers and FibroScan measurements. This facilitates a more tailored approach in DILI risk assessment for patients with liver disease initiating anti-tuberculosis therapy.

The observed strong predictive values of liver function markers and the utility of nomograms in the context of DILI prediction offer promising implications for clinical practice. The comprehensive predictive approach, including biochemical and elastographic parameters, allows for a more nuanced understanding of an individual’s risk profile, fostering the potential for tailored therapeutic strategies and closer monitoring to mitigate the impact of DILI. Notably, combining these assessments offers a fine-grained risk stratification method, potentially enabling clinicians to make informed decisions regarding treatment regimens, advocate for alternative therapies, or closely monitor patients at elevated risk.

Future studies should aim to validate and extend these findings to larger, more diverse patient populations. Additionally, investigating the performance of this predictive model in longitudinally assessing the risk of DILI throughout the course of anti-tuberculosis treatment could provide valuable insights into its dynamic predictive capacity [24]. Furthermore, exploring the potential integration of additional clinical and genetic factors into the existing model may enhance its predictive ability, paving the way for a more comprehensive and holistic approach to DILI risk assessment and management in patients with liver disease [16] [27].

A few limitations needed consideration as well. Firstly, the study had a modest sample size. Future validation in larger cohorts would strengthen the predictive model. Secondly, the effects of other clinical factors like nutritional status, concomitant medications, and lifestyle habits were not fully adjusted. Thirdly, the diagnostic criteria for DILI were based on laboratory parameters alone without histological evidence. Despite these limitations, our findings provide preliminary evidence for the utility of a non-invasive risk stratification approach in clinical practice.

## Conclusion

In conclusion, this prospective study demonstrates the feasibility of combining liver function indices and FibroScan measurements to predict anti-TB DILI. Further validation in larger cohorts and with histological correlation is warranted to strengthen the predictive model. Nonetheless, our findings provide preliminary evidence supporting the clinical applicability of a non-invasive risk stratification approach for optimizing the management of at-risk patients.

## Dodatak

### Contribution

Shaoqiang Wu and Qiaohua Yang contributed equally to this work.

### Conflict of interest statement

All the authors declare that they have no conflict of interest in this work.
